# Ontology-Mediated Querying with Horn Description Logics

**DOI:** 10.1007/s13218-020-00674-7

**Published:** 2020-06-21

**Authors:** Leif Sabellek

**Affiliations:** University of Bremen, Bremen, Germany

**Keywords:** Ontology-mediated querying, Horn description logics, Fine-grained data complexity, Query-by-example, Ontology-based data access

## Abstract

An ontology-mediated query (OMQ) consists of a database query paired with an ontology. When evaluated on a database, an OMQ returns not only the answers that are already in the database, but also those answers that can be obtained via logical reasoning using rules from ontology. There are many open questions regarding the complexities of problems related to OMQs. Motivated by the use of ontologies in practice, new reasoning problems which have never been considered in the context of ontologies become relevant, since they can improve the usability of ontology enriched systems. This thesis deals with various reasoning problems that emerge from ontology-mediated querying and it investigates the computational complexity of these problems. We focus on ontologies formulated in Horn description logics, which are a popular choice for ontologies in practice. In particular, the thesis gives results regarding the data complexity of OMQ evaluation by completely classifying complexity and rewritability questions for OMQs based on an EL ontology and a conjunctive query. Furthermore, the query-by-example problem, and the expressibility and verification problem in ontology-based data access are introduced and investigated.

## Introduction

In recent times, one has to manage huge amounts of data that arise from multiple sources, scattered across many different databases, so data is often incomplete and of heterogeneous quality. A popular method for organizing and accessing such data is via the use of ontologies. Ontologies store background knowledge about certain domains by defining terminology and describing how different terms relate to each other. They are popular in the fields of biology and medicine, since these fields are home to large amounts of pure factual knowledge, but they are also used in data-intensive applications by large enterprises. When accessing data from a traditional relational database via an ontology, this happens under the open world assumption. Under this assumption, the facts in the database are interpreted as true, but there might be more true facts that can be derived via logical reasoning using the knowledge from the ontology. For a more extensive discussion on the use of ontologies in data management, please see the dedicated survey included in this special issue [25].

An ontology is a set of logical sentences which represent knowledge about a specific domain. If queries are posed to a database in the presence of an ontology, one usually considers the query and the ontology together as a compound query, a so-called ontology-mediated query (OMQ). When answering an OMQ, one does not simply speak of answers to the query, but of certain answers, which are all answers to the query that are logically entailed by the database and the ontology. This approach has been studied extensively, see for example [6, 11, 12]. As an example, consider the following ontology about diseases, formulated in the description logic $\mathcal {EL}$:

$$\begin{aligned} \mathsf {AlzheimerDisease}&\sqsubseteq \mathsf {DementiaDisorder}\\ \mathsf {DementiaDisorder}&\sqsubseteq \exists \; \mathsf {hasSite}.\mathsf {BrainPart}\\ \mathsf {BrainConcussion}&\sqsubseteq \exists \; \mathsf {hasSite}.\mathsf {BrainPart} \end{aligned}$$

The first rule says that the Alzheimer’s disease is a dementia disorder. The second rule says that every instance of $\mathsf {DementiaDisorder}$ is related to an instance of $\mathsf {BrainPart}$ via the binary relation $\mathsf {hasSite}$. The third rule states the same about $\mathsf {BrainConcussion}$.

A hospital’s database may include the following facts:

$$\begin{aligned}&\mathsf {hasFinding}(\mathsf {p12}, \mathsf {f345})&\mathsf {AlzheimerDisease}(\mathsf {f345})\\&\mathsf {hasFinding}(\mathsf {p45}, \mathsf {f257})&\mathsf {BrainConcussion}(\mathsf {f257}) \end{aligned}$$

Assume a doctor needs a list of all patients who have a finding located in the brain. Then the OMQ consisting of the ontology above and the query

$$\begin{aligned} q(x) \leftarrow \mathsf {hasFinding}(x,y) \wedge \mathsf {hasSite}(y,z) \wedge \mathsf {BrainPart}(z) \end{aligned}$$

returns both $\mathsf {p12}$ and $\mathsf {p45}$ as certain answers. Note that finding the certain answers to an OMQ is a logical reasoning problem, which can in general be much harder than computing the answers to a traditional query (like an SQL query) in the absence of an ontology, which is merely a model checking problem.

## Horn Description Logics

Description logics (DLs) are decidable fragments of first-order logic (FO) that have become a popular choice for formulating ontologies [3, 4]. It is notable that DLs only use unary and binary predicates, where unary predicates are called concept names and binary predicates are called roles. In the example above, $\mathsf {AlzheimerDisease}$, $\mathsf {DementiaDisorder}$, $\mathsf {BrainConcussion}$ and $\mathsf {BrainPart}$ are concept names, and $\mathsf {hasFinding}$ and $\mathsf {hasSite}$ are roles. Depending on the specific DL, different sets of operators can be used to form *concepts*, which correspond to first-order formulas with one free variable. In the example, $\exists \; \mathsf {hasSite}.\mathsf {BrainPart}$ is a concept that describes all objects which are related via the role $\mathsf {hasSite}$ to an instance of the class $\mathsf {BrainPart}$. Formally, an ontology is a set of concept inclusions of the form $C_1 \sqsubseteq C_2$, meaning that every instance of the concept $C_1$ is also an instance of $C_2$. Such concept inclusions can be seen as if-then-rules.

There is a large variety of DLs with different expressive power and complexity of reasoning. Very expressive DLs like for instance $\mathcal {SHOIQ}$ can express, among others, disjunctions of concepts (‘every cat is dead or alive’), transitivity of roles (‘if *x* is a part of *y* and *y* is a part of *z*, then *x* is a part of *z*), role hierarchies (‘if *x* is the mother of *y*, then *x* is a parent of *y*’), inverse roles (‘if *x* is the mother of *y*, then *y* is a child of *x*’), number restrictions (‘every hand has five fingers’) and can refer to concrete individuals (‘everyone knows Dave’).

Less expressive DLs like $\mathcal {EL}$ on the other hand only allow simple rules like concept name inclusion (‘every student is a person’), conjunction (if *x* is a person and *x* is female, then *x* is a woman’) and existential restrictions (‘if *x* has a mother that is a dog, then *x* is a dog’ or ‘every country has a capital city’).

The reason to consider a large variety of DLs is the trade-off between expressive power and computational complexity. The more expressive the logic, the harder the reasoning problems become. To give a rough idea: Many standard reasoning problems (like checking whether a given tuple is a certain answer to an OMQ) for expressive DLs like $\mathcal {SHOIQ}$ are $\textsc {ExpTime}$-complete [26] or of even higher complexity, while for less expressive DLs like $\mathcal {EL}$ or the DL-Lite family, they are solvable in $\textsc {PTime}$ or $\textsc {coNP}$ [2, 18]. It turns out that the complexity is crucially influenced by whether or not disjunctions are allowed. The explanation is simple: Sentences using disjunctions do not immediately allow for unique conclusions to be drawn. So while the other mentioned types of sentences can be applied in in a straightforward way, leading to a unique result, sentences with disjunction behave differently and it becomes harder to check whether a certain fact is logically implied by the ontology.

For this reason, DLs without disjunctions are investigated. These DLs are called *Horn DLs* and they are a popular choice as ontology languages. Widely used ontologies like SNOMED CT (Systematized Nomenclature of Human and Veterinary Medicine – Clinical Terms) and GALEN (Generalised architecture for languages, encyclopedia and nomenclatures in medicine) are to a great extent formulated in a Horn DL. Horn DLs enjoy nice properties, most important for answering OMQs is the universal model property: It is possible to apply the rules from the ontology in a straightforward way to obtain a (generally infinite) extension of the database (the so-called *universal model*) which contains all facts that are relevant for answering certain types of queries, so that OMQs can be answered by constructing (a finite representation of) the universal model and evaluating the query as a standard (not ontology-mediated) query on the universal model. The thesis focuses on the Horn DLs Horn-$\mathcal {ALC}$, the $\mathcal {EL}$-family and the DL-Lite family.

## Reasoning Problems and Main Results

There are many open questions regarding OMQs with Horn DLs. The thesis [24] contributes to foundational research about Horn DLs. We are concerned with pinpointing the computational complexity of several decision problems involving OMQs. We focus on two areas:

1. Get a deeper understanding of the complexities of answering Horn DL OMQs.
2. Introduce new relevant reasoning problems and analyse their complexities.

We give an overview of all reasoning problems that are studied in the thesis and summarize the main results.

### Data Complexity and Rewritability of OMQs

Query answering in the presence of ontologies is a very natural problem. The input consists of an OMQ and a database and one is interested in the certain answers to the OMQ on the database. To change the question into a decision problem, one can additionally give a candidate tuple $\mathbf {a}$ of constants from the database as an input and ask whether $\mathbf {a}$ is a certain answer to the OMQ.

Interestingly, query answering is $\textsc {ExpTime}$-complete for many Horn DLs, which sounds like bad news for the usability of these logics in real-life knowledge representation scenarios. However, this result is slightly misleading because the complexity is usually measured relative to the size of the input and the database usually accounts for the biggest part of the input, while the query and the ontology are relatively small and often static. So there is a different, more refined way to measure the complexity, called *data complexity*: For every fixed OMQ, one considers the OMQ answering problem, where the input is only the database and a candidate tuple. Data complexity has been studied for many DLs [6, 11, 16, 18, 21, 23], and measured in data complexity, query answering for Horn DL OMQs is usually tractable.

With this refined view on the complexity of answering OMQs, more questions about the so-called *non-uniform data complexity* arise. One can fix an ontology language $\mathcal {L}$ and a query language $\mathcal {Q}$ and ask: What are all the possible complexities of OMQs formulated in $\mathcal {L}$ and $\mathcal {Q}$? How can OMQs that belong to the same complexity class be characterized? And the so-called *meta problem*: How complex is it to decide what the complexity of a given OMQ is? See [6, 19, 20, 28] for initial results on these questions. To classify OMQs into different complexity classes, one is interested in results of the form ‘every OMQ formulated in $\mathcal {L}$ and $\mathcal {Q}$ is either in complexity class *X* or hard for complexity class *Y*’, which shows that there are no OMQs with a complexity that lies ‘strictly between *X* and *Y*’. These so-called dichotomy results also play an important role in the complexity classification of constraint satisfaction problems (CSP), the recently proven $\textsc {PTime}$/$\textsc {NP}$ dichotomy (formerly known as the Feder-Vardi conjecture) being the most famous result from this area [10, 29]. In fact, there is a very strong connection between complexities of CSPs and the data complexity of OMQs [6].

It turns out that the data complexity of an OMQ is often related to rewritability of the OMQ into other query languages. Traditional database management systems (DBMS) based on SQL or Datalog^1^ are still popular, since these have been developed for a long time and are nowadays highly optimized. This raises the question whether traditional DBMS can be utilized for answering OMQs, even though they do not explicitly provide this functionality. One way to achieve this is by *rewriting* the OMQ *Q* into a FO query (as an abstraction of SQL) or a Datalog query *q*, which means to find a *q* such that the certain answers to *Q* are equal to the answers of *q* if executed on any database. It is not always possible to find such a rewriting *q*, since even for Horn DLs, rewritings into FO are not guaranteed to exist. But if a rewriting exists, one would certainly like to know this, to make use of the existing, very optimized DBMS. Thus, an interesting question is: Given an OMQ, is it rewritable into FO or into Datalog, or into some other relevant fragment of these? Rewritability into FO implies $\textsc {AC} ^0$ data complexity, as well as Datalog rewritability (as long as the ontology is formulated in a Horn DL). For more results on rewritability of OMQs, see [6, 7, 14, 15, 17]. In particular, we consider the fragment linear Datalog, where every rule can contain at most one atom with an IDB predicate.

The main result of the thesis regarding data complexity and rewritability of OMQs is a complete characterization of OMQs based on an $\mathcal {EL}$-ontology and a conjunctive query (CQ) as the actual query: For every such OMQ, the query answering problem is either in $\textsc {AC} ^0$ or $\textsc {NL}$-complete or $\textsc {PTime}$-complete. Also, rewritability into linear Datalog is possible if and only if the OMQ has data complexity in $\textsc {NL}$. Furthermore, we show that there is no constant upper bound on the arity of IDBs used in the rules of the linear Datalog rewritings and we show that the meta-problem for this class of OMQs is ExpTime-complete.

Additionally, we discuss the difficulties that arise when trying to generalize the results to $\mathcal {ELI}$, the extension of $\mathcal {EL}$ where inverse roles are allowed. It turns out that such a characterization for $\mathcal {ELI}$ would also give a complete characterization of the complexities of CSPs with tree duality, which is a challenging open problem in the area of CSPs.

### Query-By-Example

One of the new reasoning problems we study is called query-by-example (QBE). Imagine a user exploring a knowledge base. The user would like to formulate a query but is unable to do so since (s)he is unfamiliar with the ontology language or query language. However, the user can provide positive and negative examples from the data, i.e., data that should and data that should not be returned. The QBE problem asks: Is it possible to generalize the given examples into a query that returns at least all of the positive examples, but none of the given negative examples? In the positive case, we also want to compute such a witness query. This problem is related to machine learning research: We want to learn a query from the given examples. QBE has been suggested in [30] and has been studied for traditional databases and different query languages [1, 5, 8, 9, 13, 27]. We initiate the research on QBE for OMQs.

We focus on knowledge bases with Horn-$\mathcal {ALC}$ and $\mathcal {ELI}$ ontologies and show that the question of whether there exists a witness CQ is coNExpTime-complete for Horn-$\mathcal {ALC}$ and even undecidable for $\mathcal {ELI}$. Furthermore, we investigate the size of witness CQs in the Horn-$\mathcal {ALC}$ case and show that there are cases of knowledge bases that require witness CQs of double exponential size, and we show that double exponential size is always sufficient.

The undecidability result for $\mathcal {ELI}$ is quite surprising, that even for this rather inexpressive Horn-DL, one can already encode undecidable problems in QBE. So one lesson learnt from the results about QBE and also from the results about non-uniform data complexity is that allowing inverse rules may have a strong effect on the difficulty of a problem, regarding both the computational complexity as well as the technical challenges that arise.

### Expressiblility and Verification

In ontology-based data access (OBDA), data from multiple sources is unified using a new, global vocabulary. The relations of the new vocabulary are defined in terms of the old vocabulary using queries (called mappings) over the data sources. Additionally, the global vocabulary is enriched with an ontology [22], which means that the global vocabulary only consists of unary and binary relations in this case. Using OMQs over the global vocabulary becomes then the only intended point of data access. In the process of creating such an ontology, it might become unclear whether a certain query over the sources can be already expressed as a query over the global vocabulary, that is, whether there is an OMQ that when executed over the global vocabulary returns the same answers as the input query when executed over the data sources. If there is no such OMQ, introducing more mappings or changing the ontology might be necessary.

The expressibility problem asks, given an ontology, mappings, and a query *q* over the data sources, whether *q* can be expressed as an OMQ over the global vocabulary, i.e. whether there is a query $q_t$ over the global vocabulary which, evaluated as an OMQ with the given ontology, gives the same answers as *q* evaluated over the data sources. The verification problem asks, additionally given a candidate query $q_t$ over the global vocabulary, whether $q_t$ expresses *q*.

We study the expressibility and verification problem in the OBDA setting for several Horn DLs. We consider unions of conjunctive queries (UCQs) as source and target queries and global-as-view (GAV) mappings, which means the global vocabulary is defined in terms of UCQs over the data sources. We show that both problems are $\Pi ^p_2$-complete in DL-Lite, coNExpTime-complete between $\mathcal {EL}$ and $\mathcal {ELHI}$ when source queries are rooted, and 2-ExpTime-complete for unrestricted source queries.

## Footnotes

1 Datalog is a rule-based query language that uses conjunctive queries as rules to recursively define additional relations, so-called *intensional database predicates (IDB)*.
